# Supplementary material for: Deletion of wfs1 Impairs Oligodendrocyte Precursor Cells Dorsal Distribution and Myelination Through the wfs1-hmgcs1 Axis in Zebrafish
Source: Biology (Basel). 2026 Aug 16;15(16):1402. doi: 10.3390/biology15161402 (PMC13510151; doi:10.3390/biology15161402)
Supplement: Supplementary file 1 [file biology-15-01402-s001.zip › File S1-Transcriptome sequencing data and differentially expressed genes (DEGs) in wfs1-- zebrafish at 5 dpf/2.KEGG/wfs1KOvsWT/GSEA/wfs1KO/P53_SIGNALING_PATHWAY/P53_SIGNALING_PATHWAY(DRE04115).html]

Details for gene set P53\_SIGNALING\_PATHWAY(DRE04115)[GSEA]

|  || Dataset | deg.sample |
| Phenotype | sample.cls |
| Upregulated in class | wfs1KO |
| GeneSet | P53\_SIGNALING\_PATHWAY(DRE04115) |
| Enrichment Score (ES) | 0.29640165 |
| Normalized Enrichment Score (NES) | 1.4733784 |
| Nominal p-value | 0.0 |
| FDR q-value | 0.17248452 |
| FWER p-Value | 0.729 |
Table: GSEA Results Summary

  

Fig 1: Enrichment plot: P53\_SIGNALING\_PATHWAY(DRE04115)      
 Profile of the Running ES Score & Positions of GeneSet Members on the Rank Ordered List

  

| PROBE | DESCRIPTION (from dataset) | GENE SYMBOL | GENE\_TITLE | RANK IN GENE LIST | RANK METRIC SCORE | RUNNING ES | CORE ENRICHMENT || 1 | 100330864 | LOC100330864 |  |  | 70 | 4.818 | 0.0631 | Yes |
| 2 | 436699\_1 | zgc:92658 |  |  | 273 | 3.770 | 0.1078 | Yes |
| 3 | 561021 | si:dkey-103e21.5 |  |  | 599 | 2.768 | 0.1351 | Yes |
| 4 | 171473 | ccng1 |  |  | 658 | 2.661 | 0.1694 | Yes |
| 5 | 431763 | gadd45ab |  |  | 713 | 2.560 | 0.2024 | Yes |
| 6 | 768248 | fas |  |  | 1486 | 1.661 | 0.2006 | Yes |
| 7 | 100332237 | thbs1a |  |  | 1499 | 1.654 | 0.2226 | Yes |
| 8 | 100151416 | cdkn1a |  |  | 1627 | 1.584 | 0.2401 | Yes |
| 9 | 566604 | casp3b |  |  | 2287 | 1.253 | 0.2363 | Yes |
| 10 | 393548 | gadd45aa |  |  | 2430 | 1.197 | 0.2481 | Yes |
| 11 | 30590\_1 | tp53 |  |  | 2944 | 1.051 | 0.2462 | Yes |
| 12 | 565038 | ccnd2b |  |  | 3069 | 1.021 | 0.2561 | Yes |
| 13 | 497646 | gadd45bb |  |  | 3089 | 1.017 | 0.2693 | Yes |
| 14 | 368221 | tp73 |  |  | 3357 | 0.962 | 0.2740 | Yes |
| 15 | 266794 | ccng2 |  |  | 3422 | 0.947 | 0.2848 | Yes |
| 16 | 100034507 | cdk6 |  |  | 3458 | 0.939 | 0.2964 | Yes |
| 17 | 100136840 | serpine1 |  |  | 4631 | 0.730 | 0.2694 | No |
| 18 | 30637\_1 | mdm2 |  |  | 4722 | 0.715 | 0.2763 | No |
| 19 | 100004321 | LOC100004321 |  |  | 5124 | 0.665 | 0.2727 | No |
| 20 | 751765 | pmaip1 |  |  | 5838 | 0.604 | 0.2584 | No |
| 21 | 368909 | rrm2b |  |  | 6644 | 0.524 | 0.2402 | No |
| 22 | 406304 | gadd45ba |  |  | 6674 | 0.522 | 0.2464 | No |
| 23 | 393356 | gorab |  |  | 6759 | 0.514 | 0.2507 | No |
| 24 | 402991 | gadd45ga |  |  | 6915 | 0.499 | 0.2526 | No |
| 25 | 334932 | mdm4 |  |  | 7417 | 0.459 | 0.2431 | No |
| 26 | 368316 | ccnb2 |  |  | 8799 | 0.381 | 0.2048 | No |
| 27 | 553652 | gtse1 |  |  | 8814 | 0.380 | 0.2095 | No |
| 28 | 406507 | gadd45gb.1 |  |  | 9742 | 0.319 | 0.1847 | No |
| 29 | 30188\_1 | ccne1 |  |  | 10089 | 0.297 | 0.1778 | No |
| 30 | 393162 | chek1 |  |  | 10187 | 0.291 | 0.1787 | No |
| 31 | 415165 | ccne2 |  |  | 10615 | 0.265 | 0.1689 | No |
| 32 | 336998\_1 | chek2 |  |  | 11434 | 0.220 | 0.1461 | No |
| 33 | 321875\_1 | rchy1 |  |  | 11492 | 0.217 | 0.1473 | No |
| 34 | 368415 | ptenb |  |  | 11887 | 0.196 | 0.1375 | No |
| 35 | 559425 | bida |  |  | 12011 | 0.190 | 0.1362 | No |
| 36 | 571033 | pidd1 |  |  | 12588 | 0.160 | 0.1203 | No |
| 37 | 557302 | casp8l2 |  |  | 12635 | 0.157 | 0.1210 | No |
| 38 | 100302472 | adgrb1b |  |  | 13028 | 0.139 | 0.1105 | No |
| 39 | 100049706 | casp8l1 |  |  | 13088 | 0.136 | 0.1105 | No |
| 40 | 80973 | cdk1 |  |  | 13334 | 0.124 | 0.1045 | No |
| 41 | 406715 | cdk2 |  |  | 13336 | 0.124 | 0.1061 | No |
| 42 | 114401 | bcl2l1 |  |  | 13894 | 0.097 | 0.0899 | No |
| 43 | 403079 | igfbp3 |  |  | 14265 | 0.080 | 0.0794 | No |
| 44 | 100037391 | rfwd2 |  |  | 14724 | 0.059 | 0.0658 | No |
| 45 | 58081 | baxa |  |  | 14773 | 0.057 | 0.0650 | No |
| 46 | 58025 | ccnb1 |  |  | 14783 | 0.056 | 0.0655 | No |
| 47 | 794088\_1 | ptena |  |  | 15035 | 0.044 | 0.0582 | No |
| 48 | 58022 | casp8 |  |  | 15145 | 0.039 | 0.0553 | No |
| 49 | 550597 | ei24 |  |  | 15575 | 0.018 | 0.0421 | No |
| 50 | 799608 | ccnd2a |  |  | 15743 | 0.009 | 0.0369 | No |
| 51 | 171473\_1 | ccng1 |  |  | 16200 | -0.008 | 0.0227 | No |
| 52 | 436933 | sesn1 |  |  | 16367 | -0.013 | 0.0176 | No |
| 53 | 570772 | bcl2a |  |  | 16638 | -0.021 | 0.0094 | No |
| 54 | 561901 | thbs1b |  |  | 17765 | -0.066 | -0.0251 | No |
| 55 | 393779 | rprmb |  |  | 17782 | -0.066 | -0.0247 | No |
| 56 | 567770 | atr |  |  | 18166 | -0.084 | -0.0356 | No |
| 57 | 58131 | apaf1 |  |  | 18211 | -0.086 | -0.0358 | No |
| 58 | 406840 | sesn3 |  |  | 18281 | -0.089 | -0.0368 | No |
| 59 | 394065 | ppm1da |  |  | 18368 | -0.092 | -0.0383 | No |
| 60 | 767645 | zgc:153993 |  |  | 18516 | -0.099 | -0.0415 | No |
| 61 | 80955 | siah1 |  |  | 18548 | -0.101 | -0.0411 | No |
| 62 | 571845 | siva1 |  |  | 19916 | -0.162 | -0.0819 | No |
| 63 | 373093 | hdr |  |  | 20727 | -0.200 | -0.1047 | No |
| 64 | 324098 | cd82a |  |  | 20735 | -0.200 | -0.1022 | No |
| 65 | 558619 | si:dkey-42i9.6 |  |  | 21304 | -0.230 | -0.1170 | No |
| 66 | 557507 | aifm2 |  |  | 21367 | -0.235 | -0.1157 | No |
| 67 | 30222 | ccnd1 |  |  | 21522 | -0.242 | -0.1173 | No |
| 68 | 324137 | cd82b |  |  | 22109 | -0.273 | -0.1320 | No |
| 69 | 403064 | atm |  |  | 22311 | -0.285 | -0.1345 | No |
| 70 | 559425\_1 | bida |  |  | 22391 | -0.289 | -0.1331 | No |
| 71 | 100149048 | si:zfos-80g12.1 |  |  | 24470 | -0.418 | -0.1928 | No |
| 72 | 30188 | ccne1 |  |  | 24664 | -0.426 | -0.1931 | No |
| 73 | 569515 | cdk21 |  |  | 24908 | -0.441 | -0.1947 | No |
| 74 | 492763 | casp9 |  |  | 25122 | -0.455 | -0.1953 | No |
| 75 | 415158 | cycsb |  |  | 25213 | -0.461 | -0.1918 | No |
| 76 | 100149745 | sesn2 |  |  | 25398 | -0.475 | -0.1912 | No |
| 77 | 768248\_1 | fas |  |  | 26075 | -0.536 | -0.2052 | No |
| 78 | 100004133 | ddb2 |  |  | 26394 | -0.566 | -0.2075 | No |
| 79 | 567524 | tsc2 |  |  | 26670 | -0.595 | -0.2081 | No |
| 80 | 30637 | mdm2 |  |  | 26774 | -0.606 | -0.2031 | No |
| 81 | 553689 | rprma |  |  | 26967 | -0.626 | -0.2007 | No |
| 82 | 114433 | igf1 |  |  | 27128 | -0.641 | -0.1970 | No |
| 83 | 336998 | chek2 |  |  | 27308 | -0.650 | -0.1939 | No |
| 84 | 492468\_2 | ppm1db |  |  | 27535 | -0.677 | -0.1918 | No |
| 85 | 796473 | tp53i3 |  |  | 27571 | -0.681 | -0.1837 | No |
| 86 | 449996 | ccnd3 |  |  | 27759 | -0.710 | -0.1799 | No |
| 87 | 794088\_2 | ptena |  |  | 27793 | -0.715 | -0.1713 | No |
| 88 | 494479 | perp |  |  | 27900 | -0.728 | -0.1647 | No |
| 89 | 777730 | cdk4 |  |  | 28245 | -0.777 | -0.1650 | No |
| 90 | 563527 | adgrb1a |  |  | 28307 | -0.788 | -0.1563 | No |
| 91 | 561535 | zmat3 |  |  | 28619 | -0.842 | -0.1547 | No |
| 92 | 100007048 | bcl2b |  |  | 29242 | -0.987 | -0.1608 | No |
| 93 | 561021\_1 | si:dkey-103e21.5 |  |  | 29577 | -1.086 | -0.1566 | No |
| 94 | 30733 | rrm2 |  |  | 29935 | -1.219 | -0.1513 | No |
| 95 | 100320152 | steap3 |  |  | 29967 | -1.230 | -0.1357 | No |
| 96 | 30590 | tp53 |  |  | 30325 | -1.401 | -0.1279 | No |
| 97 | 140621\_1 | casp3a |  |  | 30781 | -1.743 | -0.1186 | No |
| 98 | 796473\_1 | tp53i3 |  |  | 30882 | -1.845 | -0.0967 | No |
| 99 | 431763\_1 | gadd45ab |  |  | 31309 | -2.449 | -0.0770 | No |
| 100 | 436699 | zgc:92658 |  |  | 31626 | -3.371 | -0.0412 | No |
| 101 | 140621 | casp3a |  |  | 31691 | -3.652 | 0.0063 | No |
Table: GSEA details [plain text format]

  

Fig 2: P53\_SIGNALING\_PATHWAY(DRE04115)      
 Blue-Pink O' Gram in the Space of the Analyzed GeneSet

  

Fig 3: P53\_SIGNALING\_PATHWAY(DRE04115): Random ES distribution      
 Gene set null distribution of ES for **P53\_SIGNALING\_PATHWAY(DRE04115)**
